# Integrated Transcriptomic and Metabolomic Mining of Candidate Genes for Weevil Resistance in Pea

**DOI:** 10.3390/plants15172675

**Published:** 2026-08-31

**Authors:** Lijuan Zhang, Chang Wang, Zaoxia Niu, Jianying Lu, Yang Shao, Long Li, Zongwen Chai, Gengmei Min

**Affiliations:** 1 Institute of Crop Sciences, Gansu Academy of Agricultural Sciences, Lanzhou 730070, China; chang288@163.com (C.W.); 15719327746@163.com (Z.N.); mingengmei@gsagr.cn (G.M.); 2 Institute of Biotechnology, Gansu Academy of Agricultural Sciences, Lanzhou 730070, China; lujianying@gsagr.cn; 3 Linxia Prefecture Academy of Agricultural Sciences, Linxia 731100, China; shaoyang1201@163.com (Y.S.); muzill_198@163.com (L.L.); 4 Gansu Provincial Agrotechnical Extension Station, Lanzhou 730020, China; chzw521258@163.com

**Keywords:** *Pisum sativum* L., *Bruchus pisorum* L., Flavonoid, Lectin, Insect pest stress

## Abstract

The pea weevil (*Bruchus pisorum* L.) causes severe yield losses in cultivated pea (*Pisum sativum* L.), yet the molecular basis of host resistance remains unclear. Here, we performed integrated transcriptomic and metabolomic analyses on pods and seeds of resistant (YWD) and susceptible (LW1) pea genotypes to characterize the constitutive defence network against weevils. Extensive transcriptional and metabolic reprogramming was observed in resistant materials, with upregulation of defence pathways and suppression of primary metabolism. Pods directed phenylpropanoid flux toward isoflavonoid biosynthesis and enhanced cutin/wax pathways, while seeds redirected flux to flavone/flavonol biosynthesis, accumulating high levels of flavone C-glycosides including isovitexin (5260.46-fold) and vitexin (3771.09-fold). We identified *Dof21* as a key transcription factor putatively regulating flavonoid biosynthesis, with its Dof domain predicted to bind promoters of *4CL1*, *CHS3* and *CYP75B*. A seed-specific lectin gene *LG16* showed 8.99-fold higher expression in resistant seeds with negligible expression in pods. The ABA catabolite dihydrophaseic acid and semi-volatile compounds vanilloloside and cimidahurinine were significantly enriched in resistant seeds, implicating ABA turnover and glycosylation in defence compound stabilization. This study provides a multi-dimensional molecular framework for weevil resistance in pea and offers key candidate genes including *Dof21*, *LG16*, *4CL1*, *CHS3* and *CYP75B* for resistant germplasm screening and marker-assisted breeding.

## 1. Introduction

Cultivated pea (*Pisum sativum* L.) is a globally important food and forage legume crop. Its seeds are rich in protein, starch, dietary fiber, mineral elements and various bioactive compounds, making it a key component of plant-based protein supply and healthy dietary patterns [1,2]. As a typical legume, pea also contributes to sustainable agricultural production through biological nitrogen fixation, soil fertility improvement and reduced nitrogen fertilizer input [2,3]. However, during pea cultivation, weevil pests cause severe damage, leading to reduced seed weight, lower germination rates, impaired appearance, and compromised processing and consumption quality [4,5,6]. Weevils (Coleoptera: Chrysomelidae: *Bruchinae*) are major insect pests of legume seeds. Among them, the pea weevil (*Bruchus pisorum* L.) is the most destructive species, causing yield losses of up to 60% annually under field conditions [7,8]. Typically, adult females oviposit on the surface of pea pods; upon hatching, larvae bore through the pod wall and enter the developing seeds, where they complete most of their development by feeding internally [9]. This cryptic feeding habit makes larvae difficult to control once inside the seed, as they become largely insulated from environmental factors and conventional management practices. Current control strategies rely heavily on chemical insecticides, fumigants and post-harvest storage measures, but long-term chemical use can lead to resistance, pesticide residues, environmental pollution and food safety risks [10,11]. Therefore, identifying and utilizing endogenous insect-resistant germplasm resources, elucidating the underlying molecular mechanisms, and breeding new varieties with enhanced resistance to weevils represent a promising pathway for green pest management and sustainable pea production.

Plants employ diverse and complex defence mechanisms against herbivorous insects, including physical barriers (e.g., morphological structures), secondary metabolites, proteinaceous defence factors, and hormone-mediated signaling pathways [12,13]. Previous studies have shown that traits such as seed coat thickness, epicuticular waxes, flavonoids, and insect-resistance proteins are associated with weevil resistance [7,14,15,16]. Flavonoids and isoflavonoids, as important secondary metabolites, play broad roles in disease resistance, insect defence, antioxidant activity, symbiotic interactions and stress responses [17,18,19]. Specifically, flavonoids can confer insect resistance through antifeedant effects, inhibition of feeding, interference with digestive enzyme activities, disruption of redox homeostasis, and suppression of larval development [19]. For instance, luteolin, apigenin, kaempferol and quercetin have been reported to influence insect feeding preferences and larval growth [20,21]. Flavone C-glycosides such as isovitexin and vitexin, owing to the stability of their C-C bonds against hydrolysis, may accumulate persistently in plant tissues and provide durable chemical defences [22,23]. Lectins constitute one of the most important classes of insecticidal proteins. When ingested by insects, lectins bind to glycoproteins on the surface of midgut epithelial cells, thereby interfering with digestion and absorption, disrupting gut integrity, or impairing nutrient utilization, ultimately inhibiting insect growth and development [24].

Recent advances in multi-omics technologies offer new avenues for dissecting complex insect-resistance traits in crops. Transcriptomics can reveal gene expression reprogramming and regulatory network changes during defence responses, while metabolomics directly reflects the accumulation status of defensive metabolites. Integrated transcriptomic and metabolomic analyses help establish associations between genes, pathways and metabolites [25,26,27]. Although preliminary insights into the molecular basis of weevil resistance in pea have been gained—for example, via QTL mapping or single-tissue transcriptome sequencing that identified candidate genes related to seed coat resistance or secondary metabolism [28,29]—a comprehensive multi-omics investigation of the coordinated pod–seed defence mechanism and tissue-specific defence factors is still lacking. In this study, we performed integrated transcriptome and widely targeted metabolome analyses on pods and seeds of resistant (YWD) and susceptible (LW1) pea genotypes, combined with identification of lectin family genes, transcription factor regulatory network prediction, and RT-qPCR validation, to systematically characterize the constitutive defence mechanisms against weevils in pea. Our findings provide new evidence for understanding the molecular basis of pea resistance to weevils and offer candidate genes and theoretical foundations for screening resistant germplasm and breeding weevil-resistant pea varieties.

## 2. Results

### 2.1. Phenotypic Characterization and Insect-Resistance Evaluation of Pea Varieties LW1 and YWD

**Figure 1:**
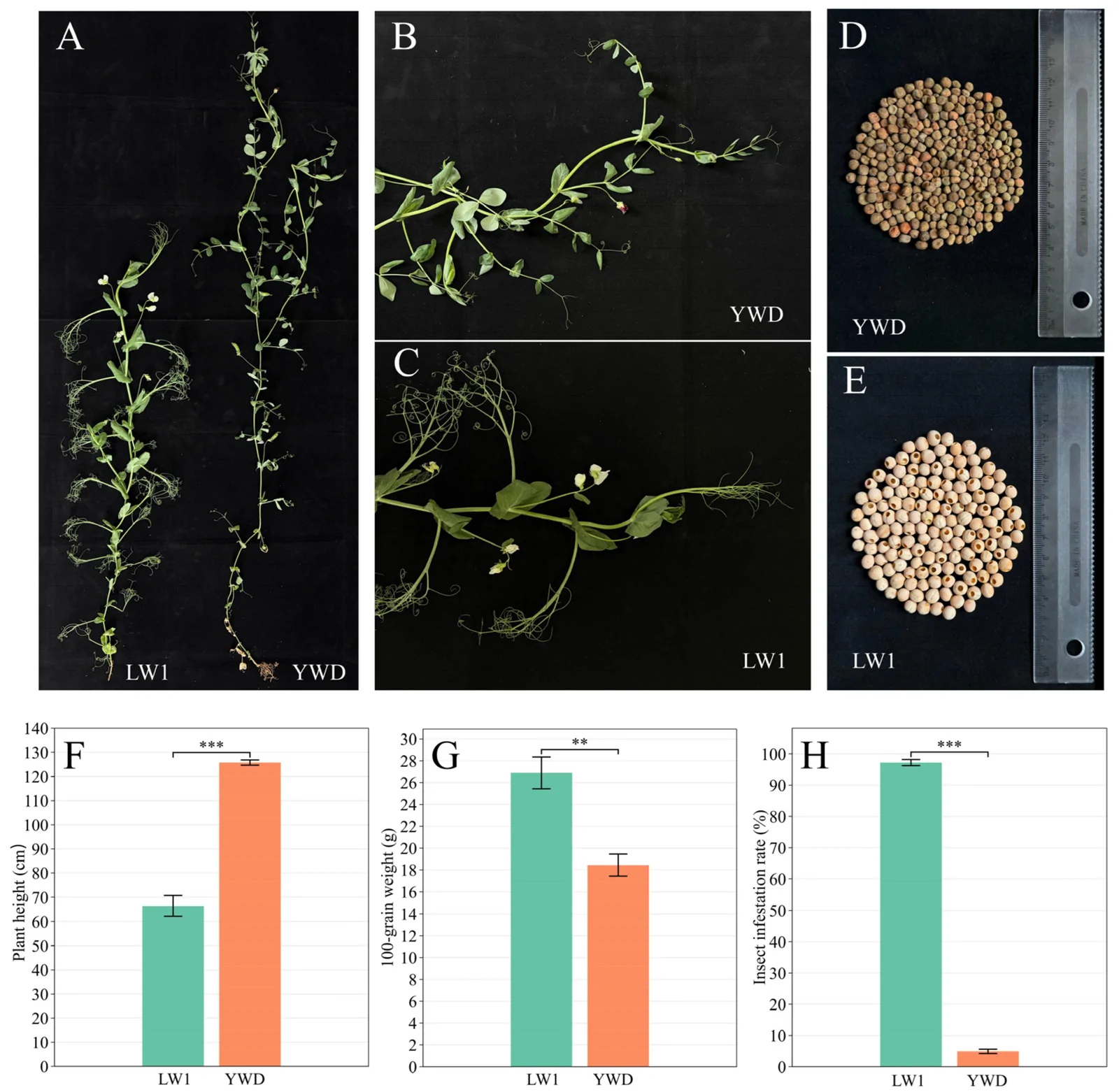
Phenotypic characterization and insect-resistance evaluation of pea varieties YWD and LW1. (**A**) Overall shoot morphology. (**B**) Leaf and flower morphology of YWD. (**C**) Leaf and flower morphology of LW1. (**D**) Seed phenotype of YWD. (**E**) Seed phenotype of LW1. (**F**) Statistical analysis of plant height. (**G**) Statistical analysis of 100-grain weight. (**H**) Statistical analysis of insect infestation rate. Statistical analyses were performed using independent samples Student’s *t*-test; ** indicates *p* < 0.01, and *** *p* < 0.001.

To characterize the phenotypic differences and weevil resistance levels of the two genotypes, we evaluated agronomic traits and insect damage in LW1 and YWD (Figure 1). The two varieties differed markedly in overall plant architecture and growth vigor (Figure 1A). YWD exhibited a normal leaf type with red flowers (Figure 1B), whereas LW1 showed a semi-leafless phenotype with white flowers (Figure 1C). Both varieties produced round seeds, but YWD seeds were mottled green (Figure 1D) while LW1 seeds were white. Quantitative measurements revealed that YWD attained a mean plant height of 125.79 cm, significantly exceeding the 66.45 cm recorded for LW1 (*p* < 0.001) (Figure 1E). However, the susceptible variety LW1 produced heavier seeds, with a 100-grain weight of 26.89 g versus 18.44 g for YWD (*p* < 0.01) (Figure 1F). Under natural field infestation, LW1 seeds were nearly completely damaged, with a seed infestation rate of 97.26%, whereas only 4.91% of YWD seeds were infested (Figure 1D,E,G). These results demonstrate that LW1 and YWD differ substantially in both growth characteristics and weevil resistance, making YWD an ideal material for dissecting the molecular mechanisms underlying pea weevil resistance.

### 2.2. Transcriptomic Characterization

**Figure 2:**
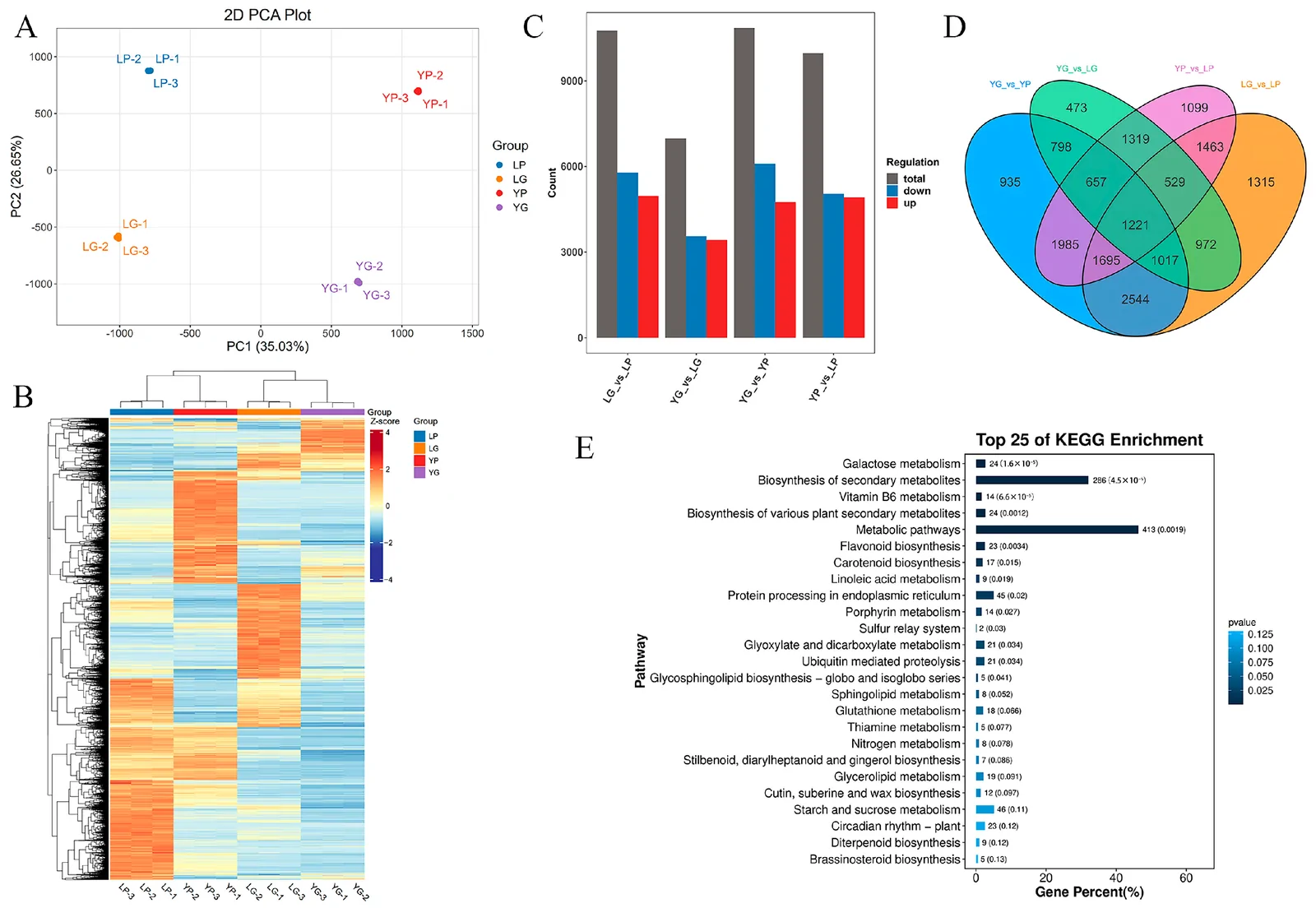
Transcriptomic characterization. (**A**) PCA plot computed with log2-transformed FPKM values of all genes. (**B**) Expression profile heatmap of all differentially expressed genes, generated using Z-score-normalized FPKM values; gene and sample dendrograms indicate expression profile similarity. (**C**) Statistics of DEG numbers across different groups. (**D**) Venn diagram of DEGs among different comparison groups. (**E**) KEGG pathway enrichment analysis of upregulated DEGs in the YG vs. LG comparison.

We performed transcriptome sequencing on pods and seeds of both susceptible and resistant pea genotypes, generating four sample groups, namely resistant pods (YP), resistant seeds (YG), susceptible pods (LP), and susceptible seeds (LG). Sequencing results are summarized in Table S1. Principal component analysis (Figure 2A) showed tight clustering of all three biological replicates per group, confirming good reproducibility and reliability of the data. PC1 and PC2 explained 35.03% and 26.65% of the total variance, respectively. Along PC1, samples separated clearly by genotype, with resistant materials YG and YP clustering on the positive side and susceptible materials LG and LP on the negative side. Along PC2, samples separated by tissue type, with seeds YG and LG on the positive side and pods YP and LP on the negative side. Hierarchical clustering of the expression profiles (Figure 2B) corroborated this pattern, showing YG clustered with LG and YP clustered with LP and confirming that tissue identity is the dominant source of transcriptomic variation.

To identify gene expression differences among materials and tissues, we analyzed differentially expressed genes (DEGs), across four comparison groups, namely YG versus YP, LG versus LP, YG versus LG, and YP versus LP, as shown in Figure 2C. In genotype comparisons, 6986 DEGs were identified between YG and LG, of which 3556 were downregulated and 3430 upregulated, while 9968 DEGs were identified between YP and LP, of which 5045 were downregulated and 4923 upregulated. Tissue comparisons, namely YG versus YP and LG versus LP, each yielded over 10,000 DEGs, indicating substantial transcriptomic divergence between seeds and pods during development. Venn diagram analysis shown in Figure 2D revealed that 1221 DEGs were shared among all four comparison groups. These genes are likely involved primarily in organ differentiation between seeds and pods, as their differential expression is common to both resistant and susceptible genotypes.

To further explore the biological functions of the DEGs, we performed KEGG pathway enrichment analysis on significantly up- and downregulated DEGs across the four comparisons (Figure 2E and Figure S1). In YG versus LG (Figure 2E), upregulated DEGs were enriched in “flavonoid biosynthesis”, “protein processing in endoplasmic reticulum”, “linoleic acid metabolism”, and “glutathione metabolism”. Conversely, downregulated DEGs in YG versus LG (Figure S1A) were predominantly associated with basal metabolic pathways, including “glycolysis/gluconeogenesis”, “carbon metabolism”, and “starch and sucrose metabolism”. In YP versus LP (Figure S1B), genes involved in “photosynthesis—antenna proteins”, “starch and sucrose metabolism”, and “porphyrin metabolism” were significantly downregulated. In YP versus LP (Figure S1C), upregulated genes were significantly enriched in “isoflavonoid biosynthesis”, “phenylpropanoid biosynthesis”, and “flavonoid biosynthesis”. “Cutin, suberine and wax biosynthesis” was also enriched. In susceptible material (LG versus LP, Figure S1D,E), seeds showed significant downregulation of defence-related pathways, including “plant–pathogen interaction” and “plant hormone signal transduction”; in particular, “flavonoid biosynthesis”, “isoflavonoid biosynthesis”, and “phenylpropanoid biosynthesis” were all downregulated (Figure S1D). In contrast, growth-associated processes, including “glycolysis/gluconeogenesis”, “fatty acid biosynthesis”, and “protein export”, were overrepresented among the upregulated DEGs (Figure S1E). In YG versus YP (Figure S1F), upregulated genes were enriched in “isoflavonoid biosynthesis”, “phenylpropanoid biosynthesis”, “flavonoid biosynthesis”, and “cutin, suberine and wax biosynthesis”. Meanwhile, downregulated genes in YG versus YP (Figure S1G) were enriched in “MAPK signaling pathway—plant”, “plant hormone signal transduction”, and “phenylpropanoid biosynthesis”.

### 2.3. Metabolomic Profiling

**Figure 3:**
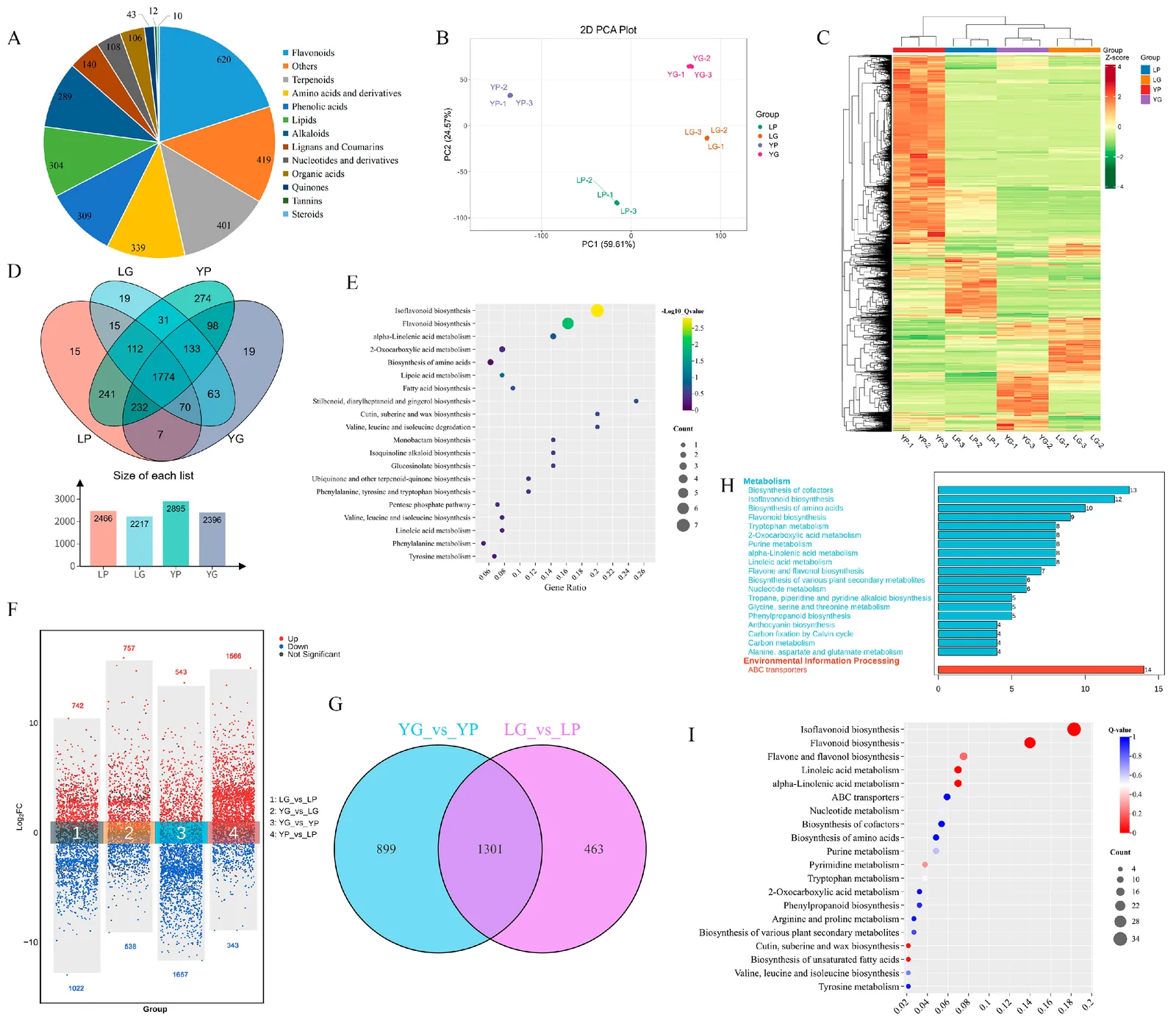
Metabolomic characterization. (**A**) Classification of identified metabolites. (**B**) PCA plot computed with log2-transformed peak area values of all metabolites. (**C**) Accumulation profile heatmap of all differentially accumulated metabolites, generated using Z-score-normalized peak area values; row and column dendrograms indicate accumulation profile similarity among metabolites and samples, respectively. (**D**) The number of metabolites detected in the four samples. (**E**) KEGG enrichment pathways of YP-specific metabolites. (**F**) Volcano plots of DEMs across four comparison groups. (**G**) Venn diagram of DEMs between the YG vs. YP and LG vs. LP comparison groups. (**H**) KEGG pathway enrichment analysis of DEMs unique to the YG vs. YP comparison. (**I**) KEGG pathway enrichment analysis of significantly upregulated metabolites in the YP vs. LP comparison.

To further elucidate the biochemical basis of pea weevil resistance at the metabolite level, we performed widely targeted metabolomic analysis on the same sample sets, namely LP, LG, YP, and YG. A total of 3100 compounds were detected across the four groups, comprising 620 flavonoids, 419 unclassified metabolites, 401 terpenoids, 339 amino acids and derivatives, 309 phenolic acids, 304 lipids, 289 alkaloids, and 419 other compounds including lignans and coumarins (Figure 3A). Principal component analysis (Figure 3B) showed that PC1 and PC2 together explained 84.18% of the total variance. PC1 separated samples primarily by tissue type, with seeds of both genotypes on the positive side and pods on the negative side. PC2 separated samples by genotype, with resistant materials on the positive side and susceptible materials on the negative side. Notably, YP was more strongly separated from the seed cluster along PC1 than LP was, suggesting that YP may possess a more distinctive metabolic profile. Consistent with the DEM accumulation heatmap (Figure 3C), LP clustered with LG and YG, displaying an accumulation pattern distinct from that of YP. To explore YP-specific metabolic features, we examined the number of metabolites detected in each of the four groups. The results showed that 2466, 2217, and 2396 metabolites were detected in LP, LG, and YG, respectively, whereas YP yielded 2895 metabolites, substantially more than the other three groups (Figure 3D). Among these, 274 metabolites were unique to YP, and enrichment analysis revealed that these metabolites were predominantly involved in isoflavonoid biosynthesis and flavonoid biosynthesis (Figure 3E).

Differential metabolite analysis (Figure 3F) identified 1295 DEMs between YG and LG, of which 757 were significantly upregulated and 538 downregulated in resistant seeds. A total of 1909 DEMs were identified between YP and LP, with 1566 upregulated and 343 downregulated. Notably, in both seeds and pods of the resistant genotype, the number of upregulated metabolites substantially exceeded that of downregulated ones. Furthermore, 1301 DEMs were shared between the YG vs. YP and LG vs. LP comparisons, while the resistant genotype comparison (YG vs. YP) harbored 899 unique DEMs, which is far more than the 463 unique DEMs in the susceptible genotype comparison (LG vs. LP) (Figure 3G). This indicates that resistant plants undergo more extensive and specific metabolic remodeling during organ development. To investigate the biological functions of these unique DEMs, we performed KEGG pathway annotation and enrichment analysis on the 899 DEMs specific to the resistant genotype. The results (Figure 3H) showed significant enrichment in biosynthesis of cofactors, isoflavonoid biosynthesis, biosynthesis of amino acids, and flavonoid biosynthesis.

To further characterize the metabolic features associated with resistance, we performed KEGG enrichment analysis on significantly accumulated and depleted DEMs across the four comparison groups (Figure 3I and Figure S2). In YP versus LP, upregulated DEMs were significantly enriched in isoflavonoid biosynthesis, flavonoid biosynthesis, and cutin, suberine and wax biosynthesis (Figure 3I), whereas downregulated DEMs were significantly enriched in basal vegetative growth pathways, including biosynthesis of amino acids, aminoacyl–tRNA biosynthesis, and starch and sucrose metabolism (Figure S2A). In YG vs. LG (Figure S2B), four of the top five enriched pathways among upregulated DEMs belonged to phenylpropanoid metabolism, namely isoflavonoid biosynthesis, flavone and flavonol biosynthesis, anthocyanin biosynthesis, and flavonoid biosynthesis, suggesting that flavonoids may be the principal secondary metabolites contributing to insect resistance in resistant seeds. Conversely, downregulated DEMs in YG vs. LG were enriched in basal metabolic pathways, including tryptophan metabolism, plant hormone signal transduction, and several amino acid metabolism pathways (Figure S2C). In YG vs. YP, downregulated DEMs were enriched in isoflavonoid biosynthesis, linoleic acid metabolism, alpha-linolenic acid metabolism, and cutin, suberine and wax biosynthesis (Figure S2D), whereas upregulated DEMs were significantly enriched in ABC transporters, flavone and flavonol biosynthesis, arginine and proline metabolism, glutathione metabolism, and anthocyanin biosynthesis (Figure S2E). In LG vs. LP, upregulated DEMs were mainly enriched in zeatin biosynthesis and arginine and proline metabolism (Figure S2F), while downregulated DEMs were enriched in isoflavonoid biosynthesis, pantothenate and CoA biosynthesis, and flavone and flavonol biosynthesis (Figure S2G).

### 2.4. Reconstruction of the Phenylpropanoid and Downstream Metabolic Pathways

**Figure 4:**
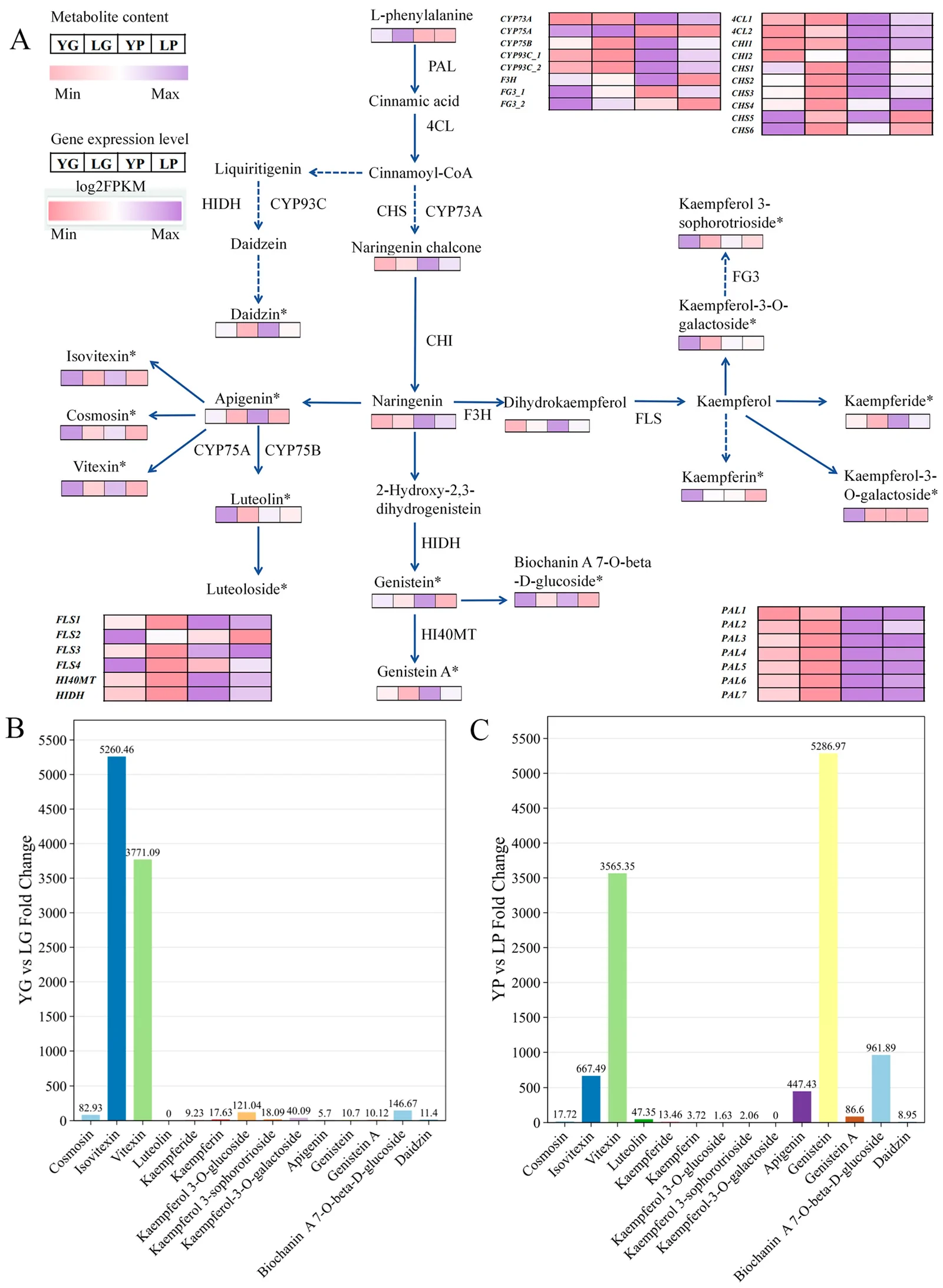
Phenylpropanoid metabolic pathway and fold changes of major phenylpropanoid differential metabolites. Solid lines indicate direct upstream–downstream relationships, while dashed lines indicate that other intermediates exist between the connected compounds. * denotes compounds that have been previously reported to possess potential insect-resistant properties. (**A**) Schematic representation of the phenylpropanoid metabolic pathway. (**B**) Fold changes of differentially accumulated metabolites in resistant seeds. (**C**) Fold changes of differentially accumulated metabolites in resistant pods. Detailed peak area values and differential analysis of DEMs are provided in Table S2.

To gain deeper insight into the precise regulation of secondary metabolic flux in weevil-resistant pea, we reconstructed the phenylpropanoid pathway and its downstream branches starting from phenylalanine, integrating transcriptomic and metabolomic data. The results (Figure 4A) revealed a total of 18 differentially accumulated metabolites and 31 differentially expressed genes within this pathway. Seven phenylalanine ammonia-lyase genes (*PAL1–7*) showed significantly higher expression levels in YP and LP than in YG and LG. Moreover, key node genes including *4CL1*, *CHI1*, *CHS1* and *CHS3* were specifically highly expressed in YP, suggesting that pods serve as the primary active tissue for basal phenylpropanoid metabolic flux. In downstream branches of phenylalanine metabolism, pods predominantly channel carbon flux toward isoflavonoid biosynthesis. Key genes responsible for isoflavonoid synthesis, such as *CYP93C_1/2*, *HIDH* and *HI40MT*, were significantly upregulated in pods (YP, LP). Consistent with their gene expression patterns, the isoflavonoid metabolites genistein and its derivative genistein A, as well as daidzin, were markedly accumulated in pods.

Although upstream *PAL* gene expression levels in YG were lower than those in YP, we observed that certain structural gene family members in the flavone and flavonol biosynthesis pathway were specifically activated in YG, including *CHS6*, flavanone 3-hydroxylase (*F3H*), and flavonol synthases (*FLS2*, *FLS4*). In YG, flavone C-glycosides such as isovitexin, vitexin and cosmosin showed specific accumulation, whereas their levels remained low in LG, LP and YP. Concurrently, multiple kaempferol glycoside derivatives, including kaempferol 3-sophorotrioside, were also significantly accumulated in YG. Furthermore, various downstream flavonoids, including apigenin, luteolin, vitexin and isovitexin, as well as isoflavonoids such as genistein, were significantly enriched in both YG and YP.

To identify the core metabolites associated with weevil resistance, we compared the peak intensities of flavonoid compounds between resistant and susceptible materials, with full quantitative data provided in Table S2. In seeds (Figure 4B), isovitexin accumulated to a mean intensity of 94,390,908 in YG versus only 17,943 in LG (5260.46-fold enrichment), and vitexin reached 92,576,795 in YG compared with 24,549 in LG (3771.09-fold enrichment). Cosmosiin, genistein A, kaempferol 3-sophorotrioside and daidzin were upregulated 82.93-, 10.12-, 121.04- and 11.40-fold in resistant seeds, respectively. In pod tissues (Figure 4C), genistein was detected at a mean intensity of 3080,616 in YP, whereas it was undetectable in all three LP replicates. Using the one-fifth-of-minimum method, missing values were imputed as 582.68, yielding an estimated fold change of 5286.97. Similarly, vitexin and biochanin A 7-O-glucoside showed peak intensities of 16,922,328 and 868,040 in YP, with 3565.35- and 961.89-fold enrichment in resistant pods, respectively. Apigenin, genistein A and cosmosiin were upregulated 447.43-, 86.60- and 17.72-fold in YP relative to LP, respectively. Notably, luteolin was undetectable in seeds of both genotypes but showed 47.35-fold enrichment in YP compared with LP. With the exception of kaempferin and kaempferol 3-O-glucoside, whose VIP values were below 1 in both the pod and resistant-versus-susceptible pod comparisons, all other compounds had VIP values greater than 1 in both comparison groups. These results suggest that isovitexin and vitexin may contribute to resistance in both seeds and pods, whereas genistein, biochanin A 7-O-beta-D-glucoside and apigenin appear to function primarily in pod defence.

### 2.5. Transcriptional Regulatory Network of Flavonoid Biosynthesis and Identification of Core TFs

**Figure 5:**
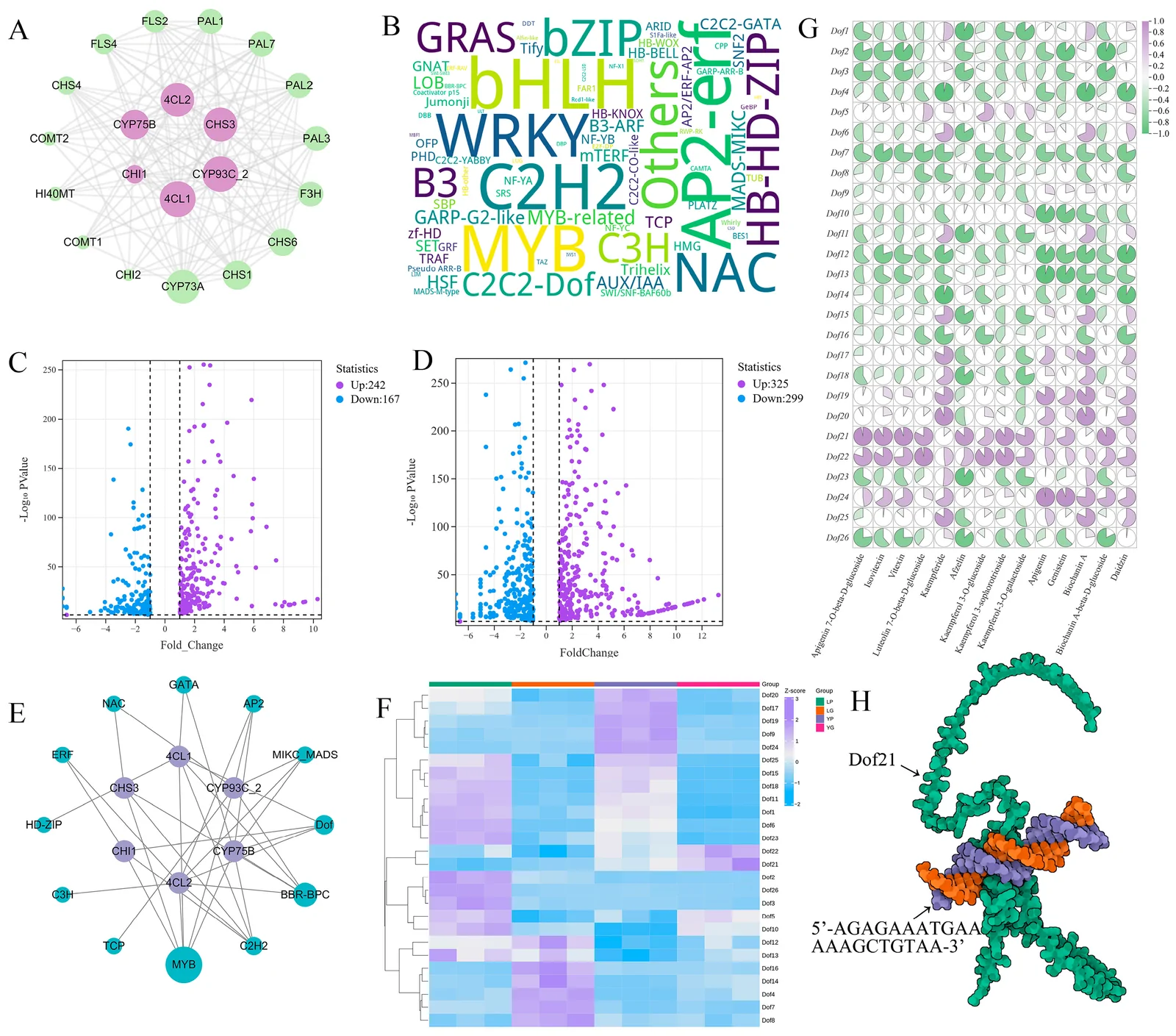
Transcription factor family identification and transcriptional regulatory analysis of flavonoid biosynthesis. (**A**) PPI network of key proteins involved in flavonoid biosynthesis; the inner circle represents the sub-network extracted using the MCODE plugin. (**B**) Identification of TF families; larger font size indicates a greater number of members. (**C**) Volcano plot of differentially expressed TFs in the YG vs. LG comparison. The dashed lines represent the cutoff for statistical significance (*p* < 0.05); points above the line are considered significantly changed. (**D**) Volcano plot of differentially expressed TFs in the YP vs. LP comparison. The dashed lines represent the cutoff for statistical significance (*p* < 0.05); points above the line are considered significantly changed. (**E**) TFBS network of key genes involved in flavonoid biosynthesis; green nodes represent TFs, purple nodes represent key genes involved in flavonoid biosynthesis, and the size of TF nodes indicates the number of connected edges. (**F**) Expression heatmap of *Dof* gene family members. (**G**) Correlation between Dof expression levels and the accumulation of key flavonoid metabolites. (**H**) Binding conformation of the Dof domain with the promoter region of *4CL1*.

To elucidate the transcriptional regulatory mechanisms underlying the massive accumulation of flavonoids in weevil-resistant pea, we performed protein–protein interaction network analysis and transcription factor binding site analysis on the differentially expressed genes involved in flavonoid biosynthesis identified above. The results (Figure 5A) revealed potential interactions among 22 proteins, with *4CL1*, *CYP93C_2*, *4CL2*, *CHS3*, *CYP75B*, and *CHI1* likely playing central roles in the interaction network. Transcription factor identification (Figure 5B) showed that 1200 TFs were co-expressed in pea seeds and pods, spanning 84 TF families including *bHLH*, *MYB*, *WRKY*, *AP2/ERF*, NAC, and *C2C2*-*Dof*. Among these, 242 TFs were significantly upregulated in YG relative to LG (Figure 5C), and 325 TFs were significantly upregulated in YP relative to LP (Figure 5D).

To further identify the TFs that may be involved in regulating flavonoid biosynthesis, we predicted TF binding sites in the promoter regions of *4CL1*, *4CL2*, *CHS3*, *CHI1*, *CYP75B*, and *CYP93C_2* and constructed a regulatory network. The results (Figure 5E) showed that the promoter regions of these core genes contained abundant binding motifs for MYB, C2H2, BBR-BPC, and Dof TFs, suggesting that these families may play important roles in flavonoid biosynthesis for insect resistance in pea. Further analysis revealed that, among the MYB, C2H2, BBR-BPC, and Dof families, the Dof family contained the largest number of differentially expressed members in both YG and YP, indicating that Dof TFs may be key regulators of flavonoid biosynthesis in the resistant genotype.

To screen for core *Dof* members, we performed expression clustering analysis on the 26 predominantly expressed *Dof* genes (Figure 5F). The results revealed tissue- and genotype-specific expression patterns among different Dof members in YG, YP, LG, and LP. *Dof20*, *Dof17*, *Dof19*, *Dof9*, and *Dof4* were mainly expressed in YP, while *Dof25*, *Dof15*, *Dof18*, *Dof6*, *Dof23*, and *Dof11* were expressed in both YP and LP. Notably, *Dof21* and Dof22 were predominantly expressed in YG and YP, with significantly higher expression levels in YG than in YP. Correlation analysis (Figure 5G) showed that the expression levels of *Dof21* and *Dof22* were highly significantly and positively correlated with the accumulation of insect-resistance-associated flavonoid metabolites. *Dof21* was upregulated 2.34-fold in YG relative to LG, while *Dof22* was upregulated 1.94-fold. However, *Dof22* showed low overall expression, with a mean FPKM of 2.01 in YG. To explore the potential regulatory role of Dof in flavonoid biosynthesis, we selected the core candidate *Dof21* and performed molecular docking simulations of its functional domain with the promoter sequences of key target genes. The results (Figure 5H and Figure S3) demonstrated that the Dof domain of *Dof21* could stably bind to the promoter regions of *4CL1*, *4CL2*, *CHS1*, *CHS3*, and *CYP75B*. These findings support the hypothesis that *Dof21* may contribute to the transcriptional activation of core flavonoid biosynthetic genes such as *4CL1* and may thereby represent one regulatory input into the biosynthesis of downstream anti-weevil defence compounds including isovitexin and genistein.

### 2.6. Volatile Metabolite Profiling and Regulatory Network of Key Anti-Insect Compounds

**Figure 6:**
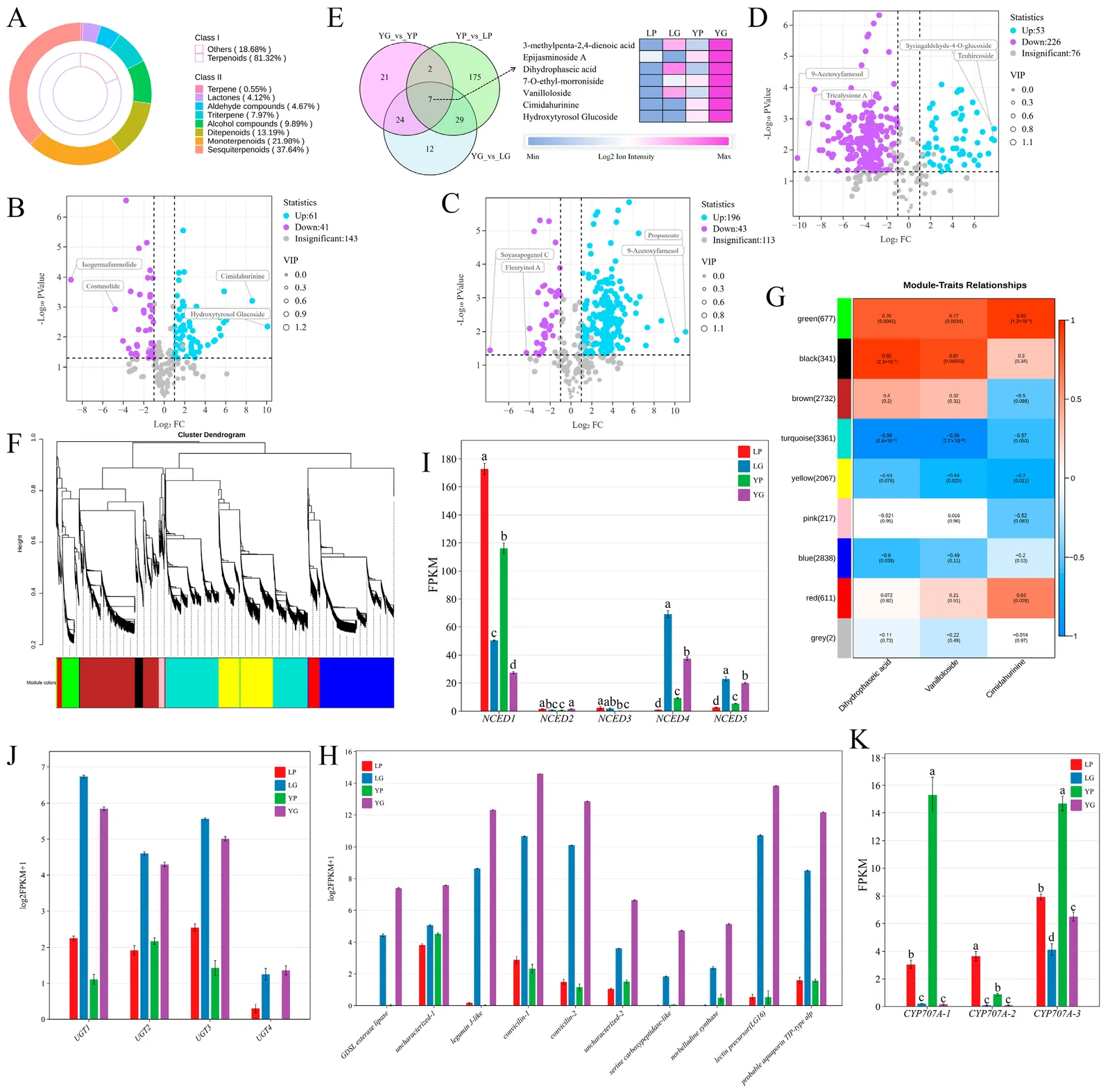
Identification of key volatile/semi-volatile compounds. (**A**) Overview of identified volatile/semi-volatile compounds. (**B**) Volcano plot of volatile/semi-volatile DEMs in the YG vs. LG comparison. (**C**) Volcano plot of volatile/semi-volatile DEMs in the YP vs. LP comparison. (**D**) Volcano plot of volatile/semi-volatile DEMs in the YG vs. YP comparison. (**E**) Venn diagram of DEMs among the YG vs. LG, YP vs. LP and YG vs. YP comparisons. (**F**) Co-expression module assignment of DEGs across the YG vs. LG, YP vs. LP and YG vs. YP comparisons. (**G**) Correlation heatmap between key volatile/semi-volatile DEMs and co-expression modules. (**H**) Expression levels of key DEGs in the module positively correlated with cimidahurinine. (**I**) Expression levels of *NCED* gene family members. (**J**) Expression levels of *UGT* gene family members. (**K**) Expression levels of *CYP707A* gene family members. In panels (**I**) and (**K**), different letters indicate significant differences determined by one-way ANOVA followed by Tukey’s HSD test (*p* < 0.05).

To investigate the role of volatile/semi-volatile compounds in pea resistance to weevils, we performed volatile profiling on seeds and pods of resistant and susceptible genotypes. The results (Figure 6A) identified a total of 364 volatile/semi-volatile compounds, of which terpenoids accounted for 81.32%. Sesquiterpenes (37.64%), monoterpenes (21.98%) and diterpenes (13.19%) were the major terpenoid subclasses. In YG vs. LG (Figure 6B), 61 metabolites were significantly upregulated and 41 downregulated, with cimidahurinine and hydroxytyrosol glucoside being notably enriched in YG. In YP vs. LP (Figure 6C), 196 metabolites were upregulated. In YG vs. YP (Figure 6D), 226 metabolites were downregulated and 53 upregulated. Notably, seven core metabolites were significantly altered across all three comparison groups (Figure 6E): 3-methylpenta-2,4-dienoic acid, epijasminoside A, dihydrophaseic acid, vanilloloside, 7-O-ethyl-morroniside, cimidahurinine and hydroxytyrosol glucoside, with all showing highest abundance in YG. This suggests that these seven compounds may serve as key defensive volatiles/semi-volatiles mediating seed resistance to weevils.

For dihydrophaseic acid, vanilloloside and cimidahurinine—compounds previously reported in the literature—we performed weighted gene co-expression network analysis (WGCNA) using transcriptomic data. The results (Figure 6F,G) showed that differentially expressed genes were partitioned into nine co-expression modules. The Green module exhibited significant positive correlations with cimidahurinine (r = 0.93, *p* = 1.2 × 10^−5^), vanilloloside (r = 0.77, *p* = 0.0034) and dihydrophaseic acid (r = 0.76, *p* = 0.0041). The Black module showed highly significant positive correlations with dihydrophaseic acid (r = 0.92, *p* = 2.3 × 10^−5^) and vanilloloside (r = 0.87, *p* = 0.00023). To further dissect the molecular basis underlying the high accumulation of these three core compounds in YG, we analysed the expression patterns of key genes involved in their metabolic pathways within the significantly positively correlated co-expression modules. *NCED* family members were identified in the module significantly correlated with dihydrophaseic acid. However, the major expressed isoforms, including *NCED1*, *NCED4*, and *NCED5*, all showed significantly higher expression in LG than in YG. We therefore examined *CYP707A*, which encodes the key enzyme catalysing ABA degradation to dihydrophaseic acid. The major expressed *CYP707A-3* isoforms showed significantly higher expression in YG than in LG. This inverse relationship between *CYP707A* expression and dihydrophaseic acid content may account for the significantly higher accumulation of dihydrophaseic acid in YG relative to LG. This suggests that resistant materials may accumulate dihydrophaseic acid through modulation of ABA turnover. In the co-expression module positively correlated with vanilloloside, *UGT1*, *UGT2* and *UGT3* showed orders-of-magnitude higher expression in seed tissues (LG, YG) than in pod tissues (LP and YP) (Figure 6J). In the module positively correlated with cimidahurinine (Figure 6H), multiple genes—including those encoding GDSL esterase/lipase, legumin J-like, convicilin and TIP-type aquaporin—were significantly more highly expressed in YG than in LP, LG and YP.

### 2.7. Identification and Expression Pattern Analysis of Lectin Family Genes

**Figure 7:**
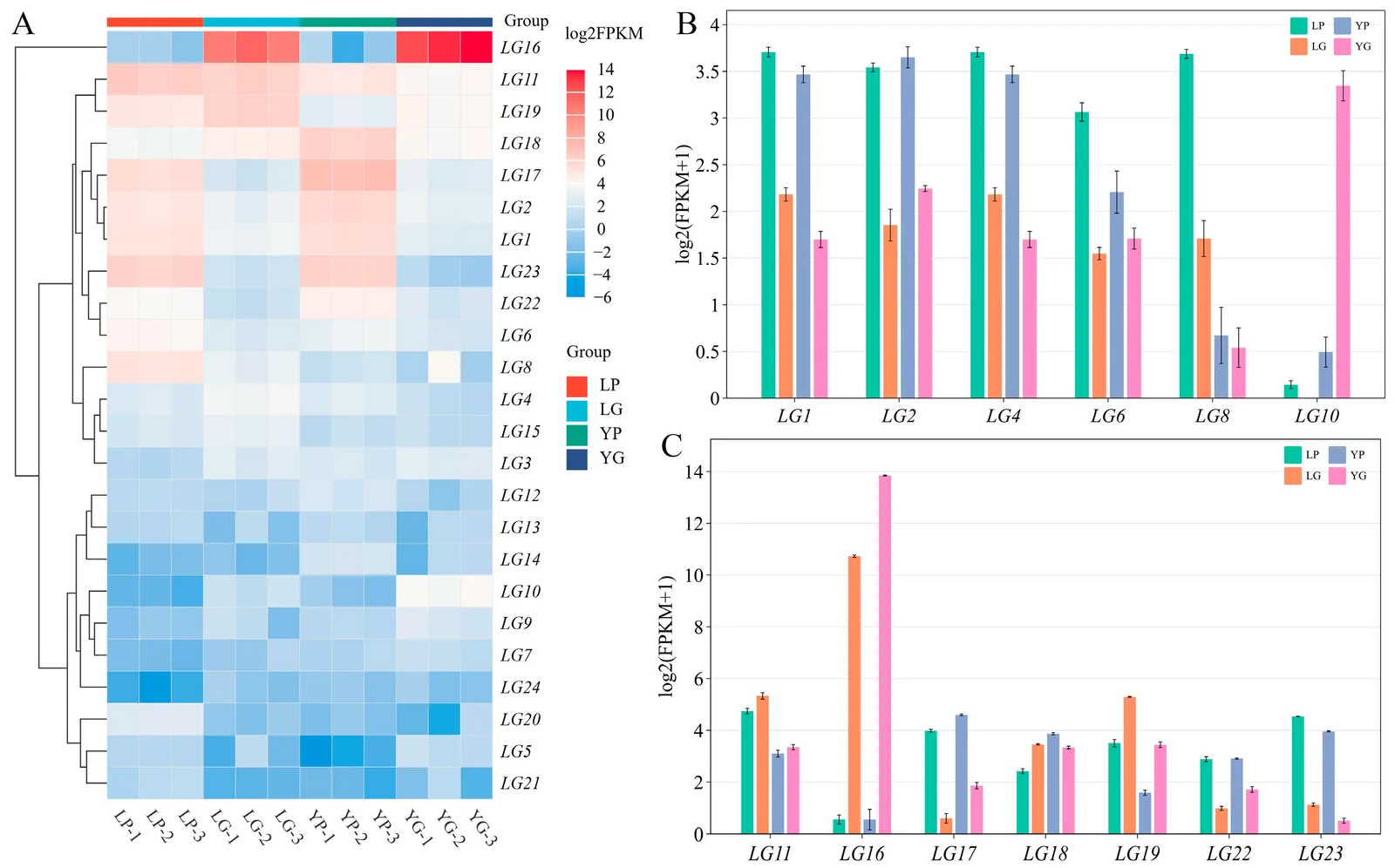
Expression analysis of lectin family genes. (**A**) Expression heatmap of the 24 lectin family genes across the four sample groups. Expression values are displayed as log_2_FPKM and row-scaled. (**B**,**C**) Expression levels of the 13 lectin genes with FPKM greater than 5 in at least one sample group, presented as log_2_(FPKM + 1) with individual data points shown. Full FPKM values and statistical comparisons are provided in Table S3. Genes with ∣log2FC∣ ≥ 1 and *p*adj < 0.05 were considered significantly differentially expressed.

In addition to secondary metabolites, insect-resistance defence proteins also play a critical role in plant defence against herbivorous insects. In this study, we identified lectin family genes in the pea transcriptome. A total of 24 lectin genes were detected in pea seeds and pods and were designated *LG1* to *LG24* (Figure 7A); full FPKM values and statistical comparisons are provided in Table S3. Among these 24 genes, 11 had FPKM values below 5 in all four sample groups and were defined as low-expression genes. The remaining 13 genes, each with an FPKM greater than 5 in at least one sample group, exhibited highly tissue- and genotype-specific expression patterns (Figure 7B,C).

*LG1*, *LG2*, *LG4*, *LG6*, *LG8*, *LG17*, *LG22*, and *LG23* were expressed at relatively higher levels in pods than in seeds of both genotypes and were classified as pod-predominant lectin genes, with no significant differences between the two pod genotypes (LP and YP; *p* < 0.05). *LG16* and *LG19* were relatively highly expressed in seeds and were classified as seed-predominant lectin genes. Notably, *LG16* was the most highly expressed lectin gene overall, reaching a mean FPKM of 14,726.21 in YG, approximately 8.99-fold higher than in LG, and was essentially undetectable in pods of both genotypes (FPKM < 1). *LG10* was a resistant-genotype-specific lectin gene, with no detectable expression in susceptible seeds or pods (FPKM < 1), and a mean FPKM of 9.20 in YG, which is significantly higher than in YP (*p* < 0.05). *LG11* was a susceptible-genotype-specific lectin gene, with significantly higher expression in LP and LG than in YP and YG (*p* < 0.05). *LG18* showed no pronounced tissue or genotype specificity, with the highest FPKM of 14.60 in YP and the lowest of 5.35 in LP. In summary, among the 24 lectin genes, *LG10* and *LG16* displayed the strongest YG-specific upregulation, and *LG16* exhibited the highest expression level of any lectin gene in resistant seeds. These two genes represent the leading candidate lectins for seed-specific defence against pea weevil.

### 2.8. RT-qPCR Validation of Core Insect-Resistance Genes

**Figure 8:**
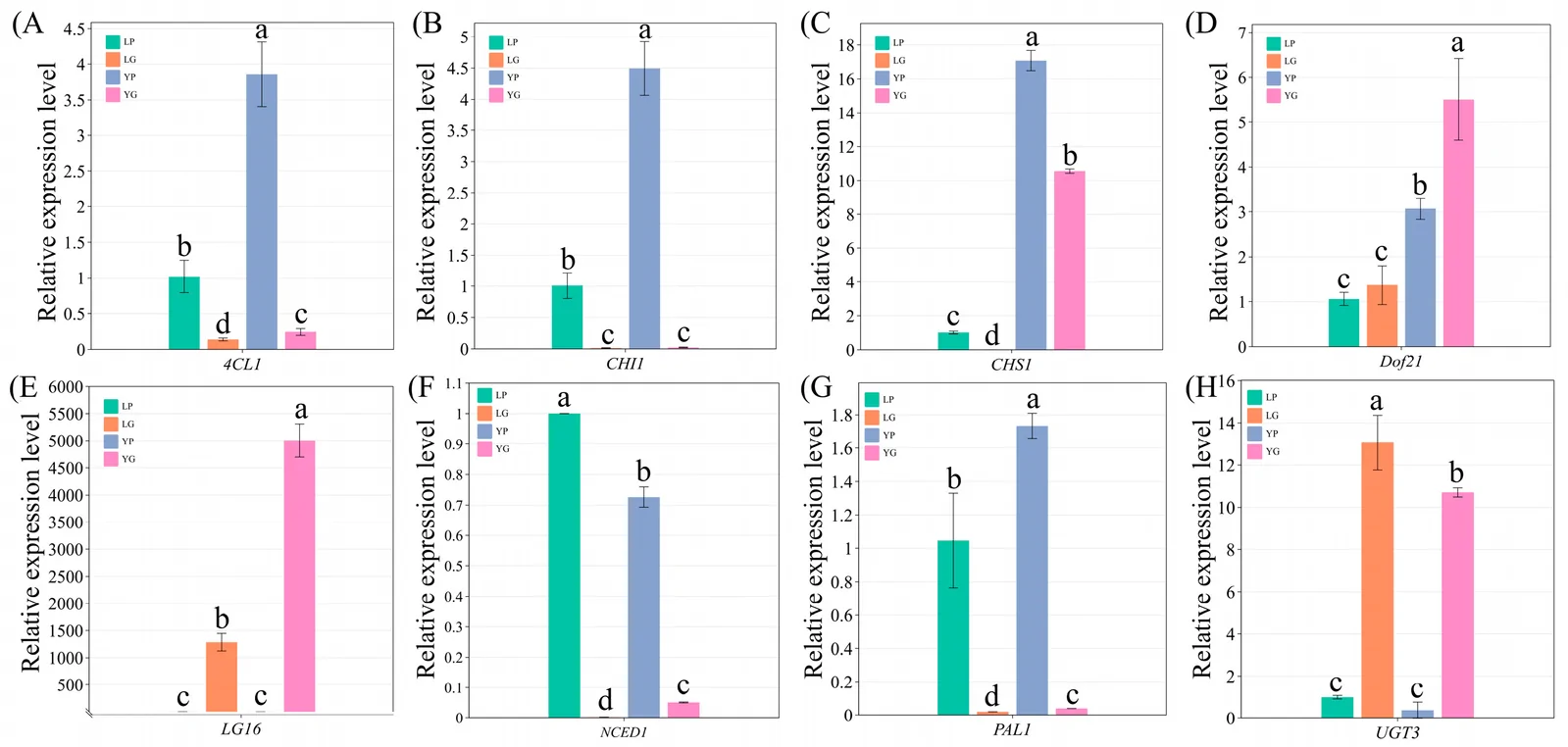
Key differentially expressed genes were validated by RT-qPCR. Relative expression was calculated using the 2^−ΔΔCT^ method with LP as the reference sample. Different letters denote statistically significant differences among groups, as determined by one-way ANOVA followed by Tukey’s HSD test at *p* < 0.05.

To verify the accuracy of the RNA-Seq data and further confirm the expression patterns of key insect-resistance genes across tissues and genotypes, we selected eight core genes for RT-qPCR analysis with LP as the reference sample, as shown in Figure 8 and Figure S4. Five structural genes involved in secondary metabolism showed excellent agreement between the two methods, with Pearson correlation coefficients of 1.00 for *4CL1*, 0.98 for *UGT3*, 0.96 for *CHI1*, 0.96 for *PAL1*, and 0.92 for *CHS1*, indicating that these genes contribute to the accumulation of secondary metabolites such as insect-resistant flavonoids. *NCED1* also displayed high consistency, with a Pearson correlation coefficient of 0.97. In contrast, two headline candidate genes showed moderate correlations, with Pearson coefficients of 0.86 for *LG16* and 0.52 for *Dof21*. Nevertheless, RT-qPCR confirmed that *LG16* was upregulated 3.89-fold in YG relative to LG; although this fold change is lower than the 8.99-fold estimate from RNA-Seq, it supports *LG16* as a major lectin-encoding gene in the resistant genotype. Similarly, RT-qPCR demonstrated that *Dof21* was upregulated 4.04-fold in YG versus LG and 3.07-fold in YP versus LP, consistent with the trends observed in the RNA-Seq analysis and supporting the potential transcriptional regulatory role of *Dof21*.

## 3. Discussion

### 3.1. Transcriptional and Metabolic Reprogramming in Resistant Materials

Plant defence against herbivorous insects typically involves large-scale transcriptional reprogramming and reallocation of metabolic resources. Because the biosynthesis of secondary metabolites and defence proteins requires substantial carbon skeletons, reducing power and energy input, plants often face a trade-off between growth and defence [30]. In this study, we found that, compared with susceptible materials, upregulated genes in resistant seeds and pods were significantly enriched in defence-related pathways, whereas genes and metabolites involved in glycolysis, carbon metabolism, amino acid biosynthesis and photosynthesis were downregulated. This suggests that resistant plants may actively sacrifice some aspects of primary growth metabolism to redirect metabolic resources toward the synthesis of insect-resistance factors such as flavonoids and lectins. This phenomenon is consistent with the induced defence mechanism in plants, whereby herbivore feeding or potential infestation triggers membrane receptor recognition, Ca^2+^ signaling, MAPK cascades, and hormonal networks involving jasmonic acid (JA) and abscisic acid (ABA), subsequently activating a large set of defence genes and secondary metabolic pathways [31]. In our study, pathways including “plant–pathogen interaction”, “plant hormone signal transduction”, “MAPK signalling pathway” and “phenylpropanoid/isoflavonoid biosynthesis” were significantly enriched in resistant seeds, indicating that resistant seeds possess a heightened constitutive or pre-activated defence state. This is particularly important for weevil pests, because once larvae burrow into the seed interior, effective mechanical or regenerative defence becomes difficult; therefore, pre-accumulation of sufficient chemical and proteinaceous defence factors before larval entry helps reduce weevil damage. In contrast, susceptible seeds showed attenuated defence-related pathways and enhanced basal growth pathways such as ribosome and glycolysis, indicating a bias toward nutrient accumulation and developmental growth.

### 3.2. Differential Defence Strategies of Pods and Seeds Against Weevils

During weevil infestation, pods serve as the first tissue encountered by ovipositing adults and penetrating larvae, whereas seeds are the primary site for prolonged larval feeding and development [32]. Thus, pods and seeds face distinct defence pressures that demand tissue-specific defence strategies. In this study, resistant pods significantly upregulated genes and metabolites in isoflavonoid biosynthesis, phenylpropanoid biosynthesis, flavonoid biosynthesis, and cutin, suberine, and wax biosynthesis pathways.

The cuticle and epicuticular waxes function as multifunctional interfaces between the plant and its environment [18,33]. Beyond their well-established roles as water-loss barriers, cuticular lipids and wax constituents directly influence insect behavior at multiple levels: surface wax composition affects insect attachment via tarsal adhesive structures, and cuticular hydrocarbons serve as contact cues mediating oviposition acceptance or rejection [34]. The co-enrichment of cutin, suberine, and wax biosynthesis pathways in YP suggests a coordinated reinforcement of the pod surface that may simultaneously impede larval penetration mechanically and alter the chemical cues perceived by ovipositing females. Similar cuticle-mediated resistance has been reported in soybean pods resistant to pod borers, where increased cuticular thickness and altered wax chemistry reduced larval entry success [35].

Furthermore, the substantial accumulation of genistein and its derivatives in pods indicates that isoflavonoids are key components of pod chemical defence. Isoflavonoids are a defining feature of legume secondary metabolism and serve as phytoalexins and constitutive defence compounds against pathogens and herbivores [36,37]. Genistein has been demonstrated to inhibit insect feeding, disrupt digestive protease activity in the midgut, and alter gut microbial communities essential for nutrient assimilation [38,39]. Beyond direct toxicity, genistein also functions as a phytoestrogenic signal that can interfere with insect moulting and metamorphosis by dysregulating ecdysteroid signaling pathways, an effect documented in lepidopteran and coleopteran systems [40]. Although the specific molecular targets of genistein may vary across insect taxa, its marked accumulation in resistant pods strongly suggests it constitutes a central chemical barrier in the pod defence network.

Unlike pods, resistant seeds showed significant accumulation of isovitexin, vitexin, luteolin, and various kaempferol glycosides, with some flavone C-glycosides reaching remarkably high levels. Flavonoids can inhibit insect growth and development through multiple mechanisms: antifeedant effects mediated by chemosensory receptor antagonism, suppression of digestive enzymes including α-amylase and trypsin, disruption of redox homeostasis through pro-oxidant activity, uncoupling of mitochondrial oxidative phosphorylation, and interference with ecdysteroid-regulated developmental transitions [17,20]. The high concentrations of flavonoids accumulated in resistant seeds may therefore impose sustained, multi-target chemical pressure on weevil larvae throughout their prolonged internal feeding period. Importantly, the dominance of flavone C-glycosides over flavonol O-glycosides in resistant seeds may have ecological significance: C-glycosidic bonds are resistant to acid and enzymatic hydrolysis, whereas O-glycosidic bonds are readily cleaved by insect gut β-glucosidases [41,42]. Consequently, flavone C-glycosides may persist intact in the insect digestive tract for longer durations, providing extended exposure and greater cumulative toxicity compared with rapidly degraded flavonol glycosides. This stability-based defence logic has been proposed for other C-glycosylated flavonoids in plant–herbivore interactions and is consistent with the evolutionary recruitment of C-glycosyltransferases in legume lineages subject to intense seed predation pressure [43,44].

### 3.3. Tissue-Specific Partitioning of Phenylpropanoid Metabolic Flux Between Pods and Seeds

Phenylpropanoid metabolism serves as a central hub for plant defence, with downstream branches including flavonoid, flavonol and isoflavonoid pathways [45]. In this study, genes involved in isoflavonoid biosynthesis—such as *PAL*, *4CL*, *CHS*, *CHI*, *CYP93C*, *HIDH* and *HI40MT*—were highly expressed in resistant pods. *CYP93C* is a key enzyme in isoflavonoid biosynthesis that converts flavanone substrates into the isoflavonoid skeleton and is critical for isoflavonoid production in legumes [45,46]. The high expression of CYP93C and its downstream genes in YP indicates that phenylpropanoid flux in pods is primarily directed toward the isoflavonoid branch, likely explaining the substantial accumulation of genistein and related isoflavonoids. In seeds, although upstream *PAL* expression was lower than in pods, flavone/flavonol branch genes such as *CHS6*, *F3H*, *FLS2*, *FLS4* and *CYP75B* were specifically highly expressed in YG, leading to massive accumulation of isovitexin, vitexin, luteolin and kaempferol glycosides. This suggests that the strategy of resistant seeds may not involve increasing total phenylpropanoid flux, but rather preferentially enhancing the content of specific flavonoid defence compounds. Notably, isovitexin and vitexin were upregulated 5260.46-fold and 3771.09-fold, respectively, in YG compared with LG. Such dramatic fold changes are unlikely to result from ordinary developmental variation and more likely represent core metabolic signatures distinguishing resistant from susceptible seeds. Previous studies have also reported higher levels of isovitexin, vitexin and luteolin in insect-resistant genotypes [47,48,49]. Moreover, both isovitexin and vitexin belong to the flavone C-glycoside class, whose C-C glycosidic bonds are stable and resistant to hydrolysis, conferring greater stability in plant tissues and insect digestive tracts [50]. For weevil larvae feeding internally over extended periods, highly stable flavone C-glycosides may serve as more persistent defence compounds than easily degradable metabolites.

### 3.4. Dof as Key Regulators of Flavonoid Metabolic Reprogramming

Tissue-specific accumulation of secondary metabolites is often orchestrated by upstream transcription factors. Flavonoid metabolism is controlled by multiple transcription factor families, including MYB, bHLH, WRKY, and Dof [51,52]. Dof transcription factors are plant-specific zinc-finger proteins that typically recognize the AAAG core motif in target gene promoters and have been functionally implicated in seed development, carbon and nitrogen metabolism, light signaling, hormone responses, and secondary metabolism [53]. In the context of plant–insect interactions, Dof TFs have been reported to mediate resistance against herbivorous insects in several systems. For instance, a Dof transcription factor in sugarcane was identified as a hub regulator of defence gene expression in response to fall armyworm herbivory [54], and Dof family members in cucumber were found to be differentially regulated upon insect challenge [55].

In this study, *Dof21* were expressed at significantly higher levels in resistant seeds and pods than in susceptible materials, and their expression levels were strongly positively correlated with the accumulation of insect-resistance-related metabolites including isovitexin, vitexin, and genistein. Molecular docking simulations predicted that the Dof domain of *Dof21* could stably interact with the promoter regions of core flavonoid biosynthetic genes, including *4CL1*, *4CL2*, *CHS1*, *CHS3*, and *CYP75B*. Although we acknowledge that molecular docking alone does not constitute proof of direct transcriptional regulation, the convergence of three independent lines of evidence (co-expression with target pathways in resistant tissues, promoter binding site enrichment, and in silico binding predictions) makes this possibility noteworthy. Further validation using EMSA, Y1H, ChIP-qPCR, or dual-luciferase reporter assays is needed to confirm the role of Dof in pea weevil resistance. Importantly, an independent line of genetic evidence supports the involvement of the DOF family in pea weevil resistance. The QTL BpSI.II on *LG7*, identified by Aznar-Fernández et al. [28] as a robust weevil resistance QTL across multiple environments, contains a DOF-type transcription factor gene (*Dolf4*) among its candidate resistance loci. Although *Dolf4* and *Dof21* are different members within the same DOF family—likely reflecting tissue-specific or regulatory-level functional divergence—their co-occurrence in independent resistance screens provides convergent genetic evidence supporting the hypothesis that DOF transcription factors are plausible central regulators of the defence transcriptome against pea weevil. The distinct tissue expression patterns of *Dof21* and DolF4 further suggest that different Dof family members may have been independently recruited to regulate defence in different anatomical contexts, a pattern consistent with the subfunctionalization model of transcription factor family evolution. Future functional characterization of *Dof21* through transgenic approaches in pea or heterologous systems will be essential to establish causality.

### 3.5. Roles of Volatile/Semi-Volatile Compounds in Seed Resistance to Weevils

Plant volatiles often play roles in insect feeding, oviposition choice, natural enemy recruitment and inter-organ signaling [56,57]. In this study, core compounds including dihydrophaseic acid, vanilloloside, cimidahurinine and hydroxytyrosol glucoside were significantly enriched in resistant seeds. Dihydrophaseic acid is a catabolite of ABA, and its accumulation suggests enhanced ABA turnover in seeds [56,57]. ABA not only regulates seed maturation, dormancy and dehydration tolerance but also interacts with JA, salicylic acid (SA) and ethylene (ET) in insect defence, exhibiting synergistic or antagonistic effects depending on insect type, tissue state and developmental stage [58,59]. Previous studies have found that increased accumulation of dihydrophaseic acid enhances plant resistance to herbivores [60]. Here, differential expression of *NCED* and *CYP707A* family genes indicated that resistant seeds may modulate ABA synthesis and degradation to maintain hormonal homeostasis, thereby influencing defence metabolite accumulation. Additionally, *UGT1*, *UGT2* and *UGT3* in the vanilloloside-correlated module were highly expressed in seeds, suggesting that UDP-glycosyltransferases may participate in stabilizing storage of phenolic or volatile precursors. UGT-mediated glycosylation alters the solubility, stability, toxicity and storage form of small-molecule metabolites, representing an important mechanism for regulating defence metabolite activity [61]. This is consistent with the accumulation of flavone C-glycosides and other glycosylated metabolites in resistant seeds, indicating that resistant seeds may broadly enhance glycosylation modifications to store defence compounds in a stable form.

### 3.6. Role of Lectin Genes in Seed Defence Against Weevils

In addition to secondary metabolites, proteinaceous defence factors constitute a major component of plant resistance to herbivorous insects. We found that the lectin gene *LG16* was dramatically upregulated in resistant seeds, while being essentially undetectable in pods of both genotypes. Lectins are carbohydrate-binding proteins that, upon ingestion by insects, bind to glycoconjugates on the surface of midgut epithelial cells. This binding can trigger a cascade of deleterious effects: interference with the peritrophic matrix that protects the midgut epithelium, disruption of microvillar architecture, inhibition of nutrient transporters, blockage of digestive enzyme secretion, and stimulation of epithelial cell apoptosis [62]. The structural basis for these effects lies in the multivalent carbohydrate recognition domains of lectins, which can cross-link multiple glycoprotein receptors and effectively “glue” the gut surface.

The insecticidal activity of plant lectins has been demonstrated against a wide range of insect orders, with Coleopteran species showing particular sensitivity to legume lectins. Snowdrop lectin, pea lectin, and Phaseolus vulgaris lectin have all been shown to exert growth-inhibitory or toxic effects on Coleoptera, Lepidoptera, and Hemiptera when expressed transgenically or delivered in artificial diets [63,64,65,66]. The molecular target in Coleoptera is primarily the heavily glycosylated peritrophic matrix and the underlying brush-border membrane of the midgut, both of which present abundant N-acetylgalactosamine and mannose residues recognized by legume lectins. The seed-specific, high-level expression of *LG16* is biologically consistent with the seed’s requirement for a pre-positioned, stable defence protein that can act immediately upon ingestion by newly hatched larvae. Unlike chemical defences that may require enzymatic activation, lectins are constitutively active proteins whose insecticidal function depends only on target glycan accessibility in the insect gut.

## 4. Materials and Methods

### 4.1. Plant Materials

The pea accessions used in this study comprised the weevil-resistant variety YWD and the susceptible variety LW1, both screened and provided by the Crop Research Institute, Gansu Academy of Agricultural Sciences. All experimental materials were uniformly cultivated in greenhouse facilities at the Gansu Academy of Agricultural Sciences under standard water and fertilizer management. For phenotypic evaluation, six biological replicates per variety were grown under natural field infestation conditions. At maturity, plant height was measured from the base of the stem to the tip of the main shoot. Seeds were harvested and air-dried to constant weight, and the 100-grain weight was determined using an electronic balance. The seed infestation rate was assessed by counting the number of seeds with visible weevil emergence holes or internal larval damage among 100 randomly selected seeds per replicate, expressed as a percentage. Significant differences between the two varieties were determined by an independent-samples t-test.

For transcriptomic and metabolomic analyses, flowers were tagged at full bloom stage, and fresh young pods and developing seeds were collected from both varieties at 20 days after flowering. This period coincides with the peak oviposition period of pea weevil and also represents the developmental stage at which first-instar larvae begin to penetrate developing seeds. To ensure representative sampling, each sample consisted of pooled tissues from five randomly selected plants of uniform growth, with three independent biological replicates. Resistant seeds and pods were designated YG and YP, respectively, and susceptible seeds and pods were designated LG and LP, respectively. All collected samples were immediately frozen in liquid nitrogen and subsequently stored at −80 °C until further use for transcriptome sequencing, metabolomic profiling, and RT-qPCR validation.

### 4.2. Methods

#### 4.2.1. Transcriptome Sequencing and Analysis

Total RNA was extracted using the CTAB method and dissolved in DEPC-treated water. RNA concentration was measured using a Qubit fluorometer, and RNA integrity was assessed with a Qsep400 high-throughput fragment analyzer to ensure sample quality. Polyadenylated mRNA was enriched using Oligo(dT) magnetic beads, fragmented, and reverse-transcribed into first-strand cDNA using random hexamer primers. During second-strand synthesis, dUTP was incorporated to enable strand-specific library construction. Libraries were then subjected to end repair, adapter ligation, PCR amplification and magnetic bead purification, with insert sizes of 250–350 bp selected. The resulting double-stranded DNA libraries were circularized to single-stranded circles and amplified via phi29 polymerase to generate DNA nanoballs (DNBs), which were sequenced on the MGI DNBSEQ-T7 platform. Raw reads were quality-filtered using fastp v0.23.2 [67] to obtain clean reads, which were aligned to the reference genome using HISAT2 v2.2.1 [68]. Transcript assembly and novel gene prediction were performed with StringTie v2.1.6 [69]. The reference genome used was that of the cultivated pea variety ‘Zhongwan6’, downloaded from the NCBI database (https://www.ncbi.nlm.nih.gov/, accession GCF_024323335.1, accessed on 13 January 2026). Gene expression levels were quantified using featureCounts and normalized as fragments per kilobase of transcript per million mapped reads (FPKM).

PCA of all samples was performed on log_2_-transformed FPKM values of all genes using the stats v3.5.1 package in R v3.5.1. Heatmaps were generated using Z-score-normalized FPKM values of all differentially expressed genes with the ComplexHeatmap v2.12.0 package in R v4.2.0 [70]. Differential expression analysis was performed using DESeq2 v1.22.1 [71] based on raw read counts. Genes with an absolute log_2_ fold change (|log_2_FC|) ≥ 1 and an adjusted *p*-value (*p*adj) < 0.05 were considered DEGs. KEGG Orthology annotation was performed using KofamScan, and KEGG pathway enrichment analysis was visualized using ggplot2 v3.3.0 [72]. Venn diagrams of differentially expressed genes among groups were generated using the Venn Diagram v1.6.20 package in R v3.5.1 with default parameters [73].

#### 4.2.2. Metabolomic Profiling and Analysis

Widely targeted metabolomic analysis was carried out on a UPLC-MS/MS detection platform (ultra-high-performance liquid chromatography coupled with tandem mass spectrometry). Sample preparation and mass spectrometric detection followed the protocol described by Qi et al. [74]. Differential metabolite selection was based on the orthogonal partial least squares discriminant analysis (OPLS-DA) model implemented in the MetaboAnalyst R package; data were log_2_-transformed and UV-scaled prior to modeling, and 200 permutation tests were conducted to prevent overfitting. For metabolites undetected in one or more groups, missing values were imputed as one-fifth of the minimum positive peak intensity across all samples for that metabolite, following the MetaboAnalyst 5.0 convention [75]. In pairwise comparisons, metabolites meeting the criteria of variable importance in projection (VIP > 1) and absolute fold change (fold change ≥ 2 or fold change ≤ 0.5) were defined as significantly differentially accumulated metabolites. Identified metabolites were annotated using the KEGG Compound database (http://www.kegg.jp/kegg/compound/, accessed on 25 February 2026) and mapped to the KEGG Pathway database (http://www.kegg.jp/kegg/pathway.html, accessed on 28 February 2026) for pathway enrichment analysis. PCA was performed on log_2_-transformed peak area values of all metabolites. Clustering heatmaps were generated using Z-score-normalized peak area values of all differentially accumulated metabolites. Venn diagrams were generated using the same methods described for transcriptomic analysis. Volcano plots of differential metabolites were generated using the ggplot2 v3.3.6 package in R v4.2.0 with default parameters.

#### 4.2.3. PPI Network, TFBS and Molecular Docking Analyses

PPI networks were constructed using STRING (https://cn.string-db.org/, accessed on 13 March 2026) and visualized with Cytoscape v3.10.3 [76]; sub-networks were extracted using the MCODE plugin. Transcription factor annotation was performed using iTAK v1.7a [77] with default parameters. TFBS prediction was carried out using PlantTFDB (http://planttfdb.cbi.pku.edu.cn/, accessed on 22 March 2026), with a significance threshold of q < 0.05, and the most significant binding site for each target gene was selected for subsequent molecular docking analysis. Conformational predictions of transcription factor binding to the selected TFBS were performed using AlphaFold3 (https://alphafoldserver.com/, accessed on 28 March 2026) [78] with default parameters; complexes with an ipTM score greater than 0.6 were considered to form stable complexes. All structural models were visualized with PyMOL 2 [79].

#### 4.2.4. WGCNA and Gene Family Identification

WGCNA was performed using the WGCNA v1.71 package in R v4.2.2, with a merge cut height of 0.25 [80]. To evaluate the function of lectins, gene family members were identified as follows: the Pfam model corresponding to lectins (PF00139) was downloaded from the Pfam database. An initial hmmsearch was performed against the pea genome with an E-value threshold of <0.01. Candidate sequences were used as input to rebuild the Pfam model using hmmbuild, followed by a second hmmsearch. Redundant sequences from the second search were removed, and the remaining candidates were submitted to NCBI-CDD (https://www.ncbi.nlm.nih.gov/cdd, accessed on 5 April 2026) for domain integrity verification.

#### 4.2.5. RT-qPCR Validation of Differentially Expressed Genes

RNA extraction was performed using the same method as for transcriptome sample preparation. RNA concentration and purity were assessed using a Nanodrop spectrophotometer, and RNA was diluted to 200 ng/μL. Reverse transcription was performed using the SweScript All-in-One RT SuperMix kit (Wuhan Servicebio Technology Co., Ltd., Wuhan, China, G3337) in a 20 μL reaction system, including genomic DNA removal, incubation at 25 °C for 5 min, 42 °C for 30 min, and 85 °C for 5 s to terminate the reaction. Quantitative PCR (qPCR) was performed in a 15 μL reaction volume containing 7.5 μL of 2× Universal Blue SYBR Green Master Mix, 1.5 μL of primer mix (2.5 μM each), 2.0 μL of cDNA template and 4.0 μL of nuclease-free water. Reactions were run on a CFX Connect real-time PCR system (Bio-Rad Laboratories, Hercules, CA, USA) using the following program: initial denaturation at 95 °C for 30 s; 40 cycles of denaturation at 95 °C for 15 s and annealing/extension at 60 °C for 30 s. Relative gene expression levels were calculated using the 2^−ΔΔCT^ method with LP as the reference sample. Primer sequences for target genes were designed using NCBI Primer-BLAST (https://ncbi.nlm.nih.gov/tools/primer-blast/index.cgi, accessed on 11 May 2026). *Actin* was used as the internal reference gene, and three biological replicates were performed. Primer information for qRT-PCR is provided in Table S3.

## 5. Conclusions

This study reveals the constitutive defence mechanisms of pea against the pea weevil through integrated transcriptomic and metabolomic profiling of resistant and susceptible genotypes. Pods primarily deter adult oviposition and larval penetration by reinforcing epicuticular wax layers and accumulating isoflavonoids such as genistein, a process associated with the coordinated expression of structural genes including *4CL1*, *4CL2*, *CHS3*, *CHI1*, and *CYP93C_1/2*. In contrast, seeds establish a durable chemical and proteinaceous defence barrier through massive accumulation of stable flavone C-glycosides, particularly isovitexin and vitexin, together with the seed-specific high expression of the lectin gene *LG16*. The flavone and flavonol branch in seeds is supported by structural genes including *CHS6*, *F3H*, *FLS2*, *FLS4*, and *CYP75B*. Among upstream regulators, *Dof21* emerges as a strong candidate TF for coordinating flavonoid metabolic reprogramming in resistant genotypes, supported by co-expression patterns, promoter binding site predictions, and molecular docking simulations, though functional validation through EMSA, Y1H, or transgenic approaches remains necessary. Additional candidate genes implicated in ABA turnover include *NCED4*, *NCED5*, and *CYP707A* family members, while *UGT1*, *UGT2*, and *UGT3* are associated with glycosylation-mediated stabilization of defence compounds. Genes encoding GDSL esterase/lipase, legumin J-like, convicilin1/2, and a probable aquaporin TIP-type were also identified as candidate contributors to volatile-related defence. We acknowledge that the resistant-versus-susceptible comparison is based on a single genotype pair; therefore, DEG or DEM differences may be partially attributable to genetic background effects unrelated to weevil resistance, such as cultivar-specific developmental timing or general divergence in secondary metabolism. Future studies employing additional resistant and susceptible genotypes or segregating populations will be important to confirm the genotype-level generalizability of these findings.

## Data Availability

The RNA sequencing data generated in this study have been deposited in the NGDC (https://ngdc.cncb.ac.cn/) database under accession number PRJCA068502.

## Supplementary Materials

The following supporting information can be downloaded at https://www.mdpi.com/article/10.3390/plants15172675/s1. Table S1. Transcriptome sequencing information. Table S2. Detailed peak area values and differential analysis of DEMs. Table S3. Full FPKM values and statistical comparisons of LGs. Table S4. RT-qPCR primer information. Figure S1.KEGG pathway enrichment analysis of DEGs in different comparison groups. (A) Downregulated DEGs in the YG vs. LG comparison. (B) Downregulated DEGs in the YP vs. LP comparison. (C) Upregulated DEGs in the YP vs. LP comparison. (D) Downregulated DEGs in the LG vs. LP comparison. (E) Upregulated DEGs in the LG vs. LP comparison. (F) Upregulated DEGs in the YG vs. YP comparison. (G) Downregulated DEGs in the YG vs. YP comparison. Figure S2. KEGG pathway enrichment analysis of DEMs in different comparison groups. (A) Downregulated DEMs in the YP vs. LP comparison. (B) Upregulated DEMs in the YG vs. LG comparison. (C) Downregulated DEMs in the YG vs. LG comparison. (D) Downregulated DEMs in the YG vs. YP comparison. (E) Upregulated DEMs in the YG vs. YP comparison. (F) Upregulated DEMs in the LG vs. LP comparison. (G) Downregulated DEMs in the LG vs. LP comparison. Figure S3. Binding conformations of the *Dof21* Dof domain with the promoter regions of core regulatory genes involved in flavonoid biosynthesis. Binding conformations of the Dof21 Dof domain with the promoter regions of core regulatory genes involved in flavonoid biosynthesis. (A) Binding conformation of the Dof domain with the promoter region of *4CL2*. (B) Binding conformation of the Dof domain with the promoter region of *CYP75B*. (C) Binding conformation of the Dof domain with the promoter region of *CHS1*. (D) Binding conformation of the Dof domain with the promoter region of *CHS3*. Figure S4. Correlation heatmap between FPKM values and relative expression levels of key DEGs.
