# Supplementary material for: Integrated Transcriptomic and Metabolomic Mining of Candidate Genes for Weevil Resistance in Pea
Source: Plants (Basel). 2026 Aug 31;15(17):2675. doi: 10.3390/plants15172675 (PMC13567257; doi:10.3390/plants15172675)
Supplement: Supplementary file 1 [file plants-15-02675-s001.zip › Figure S.pdf]

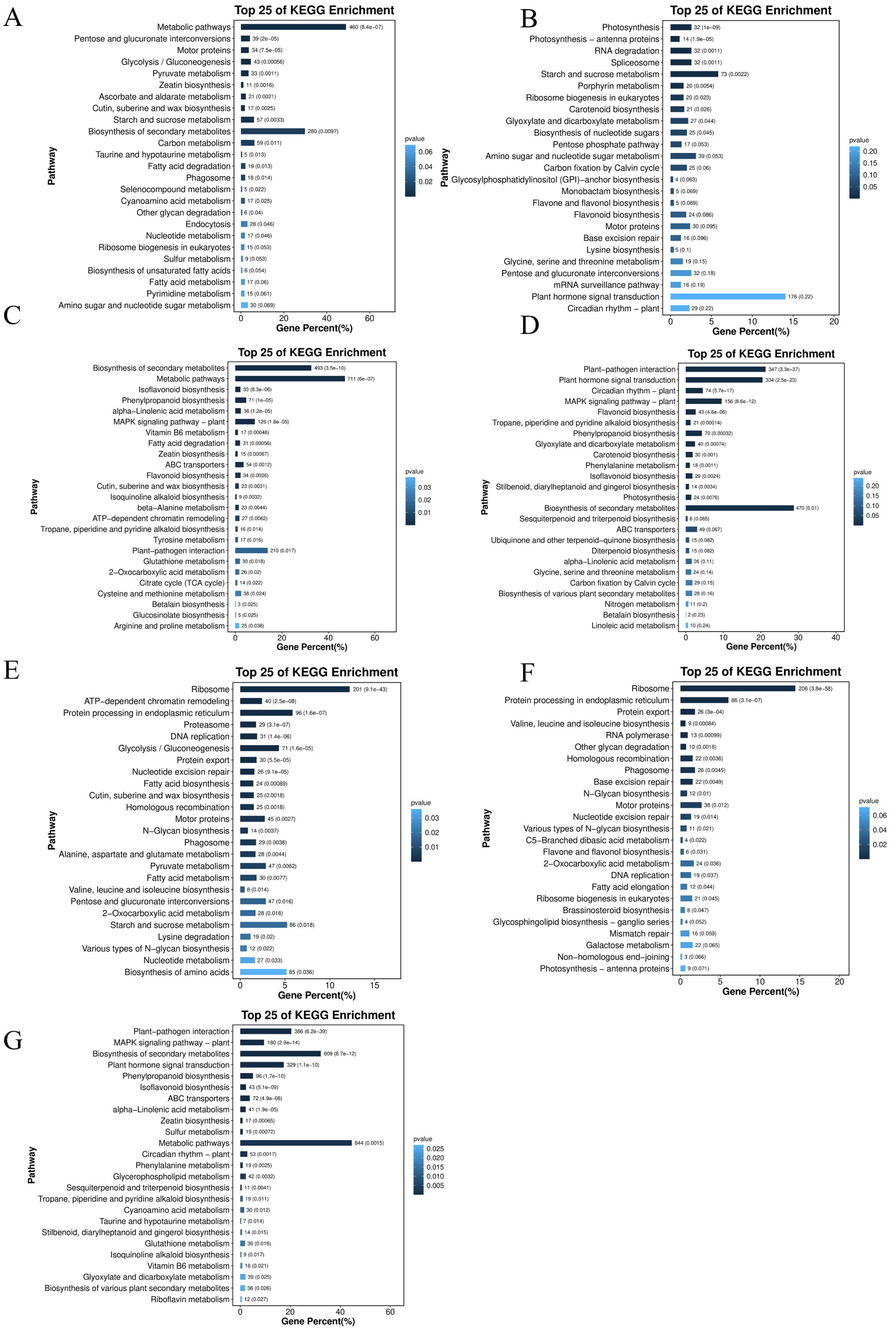

Figure S1. KEGG pathway enrichment analysis of DEGs in different comparison groups. (A) Downregulated DEGs in the YG vs LG comparison. (B) Downregulated DEGs in the YP vs LP comparison. (C) Upregulated DEGs in the YP vs LP comparison. (D) Downregulated DEGs in the LG vs LP comparison. (E) Upregulated DEGs in the LG vs LP comparison. (F) Upregulated DEGs in the YG vs YP comparison. (G) Downregulated DEGs in the YG vs YP comparison.

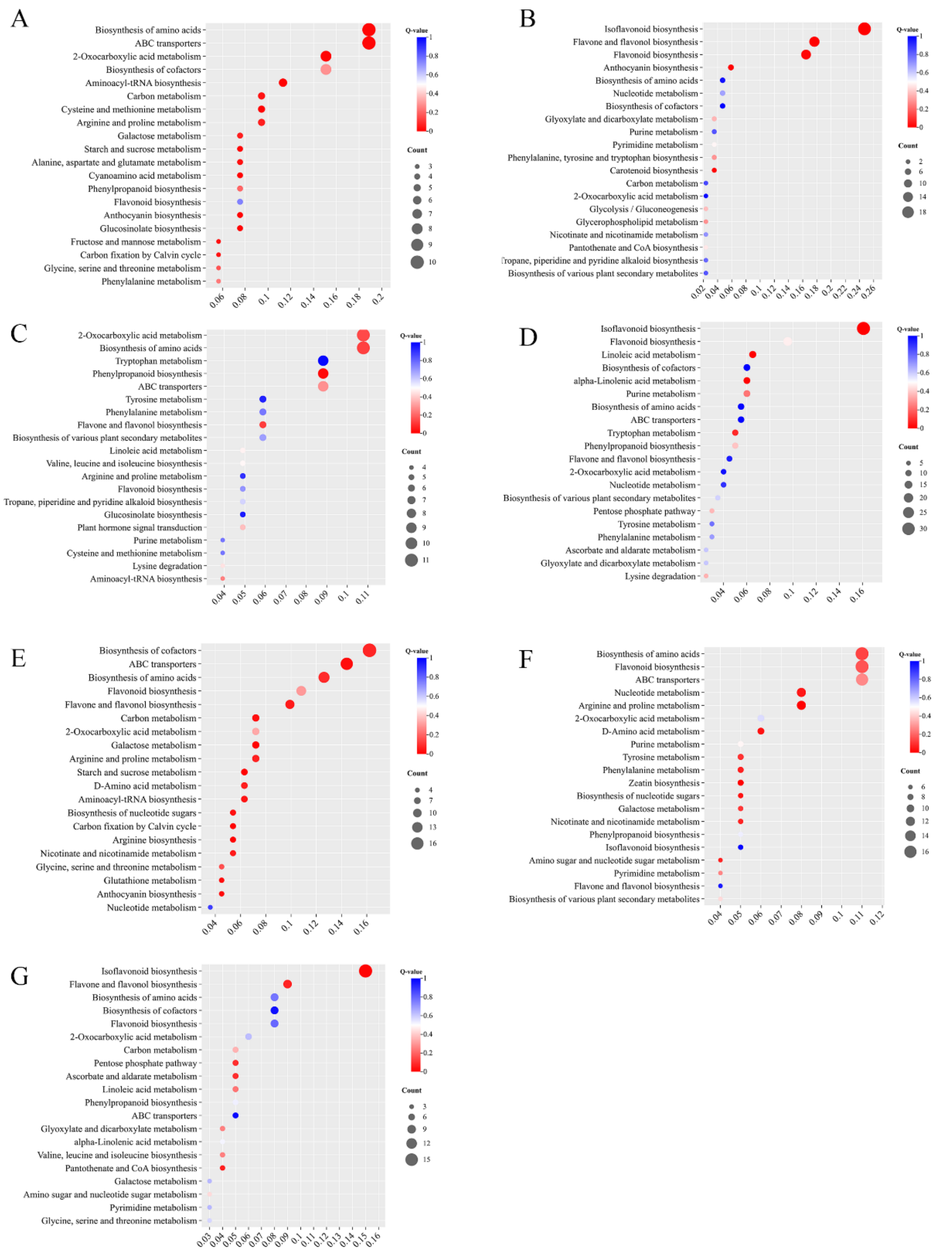

Figure S2. KEGG pathway enrichment analysis of DEMs in different comparison groups. (A) Downregulated DEMs in the YP vs LP comparison. (B) Upregulated DEMs in the YG vs LG comparison. (C) Downregulated DEMs in the YG vs LG comparison. (D) Downregulated DEMs in the YG vs YP comparison. (E) Upregulated DEMs in the YG vs YP comparison. (F) Upregulated DEMs in the LG vs LP comparison. (G) Downregulated DEMs in the LG vs LP comparison.

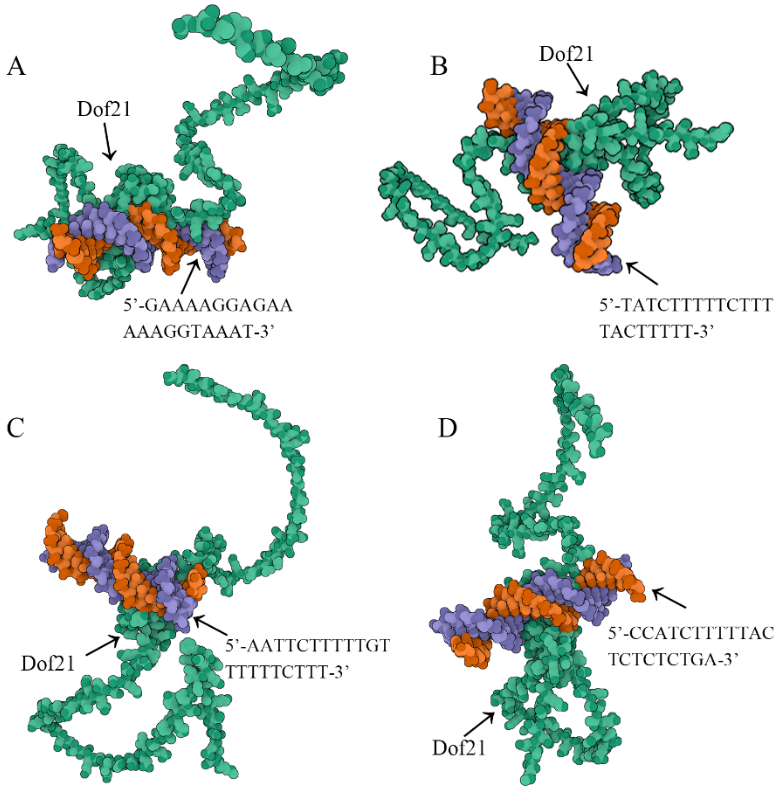

Figure S3. Binding conformations of the Dof21 Dof domain with the promoter regions of core regulatory genes involved in flavonoid biosynthesis. (A) Binding conformation of the Dof domain with the promoter region of *4CL2*. (B) Binding conformation of the Dof domain with the promoter region of *CYP75B*. (C) Binding conformation of the Dof domain with the promoter region of *CHS1*. (D) Binding conformation of the Dof domain with the promoter region of *CHS3*.

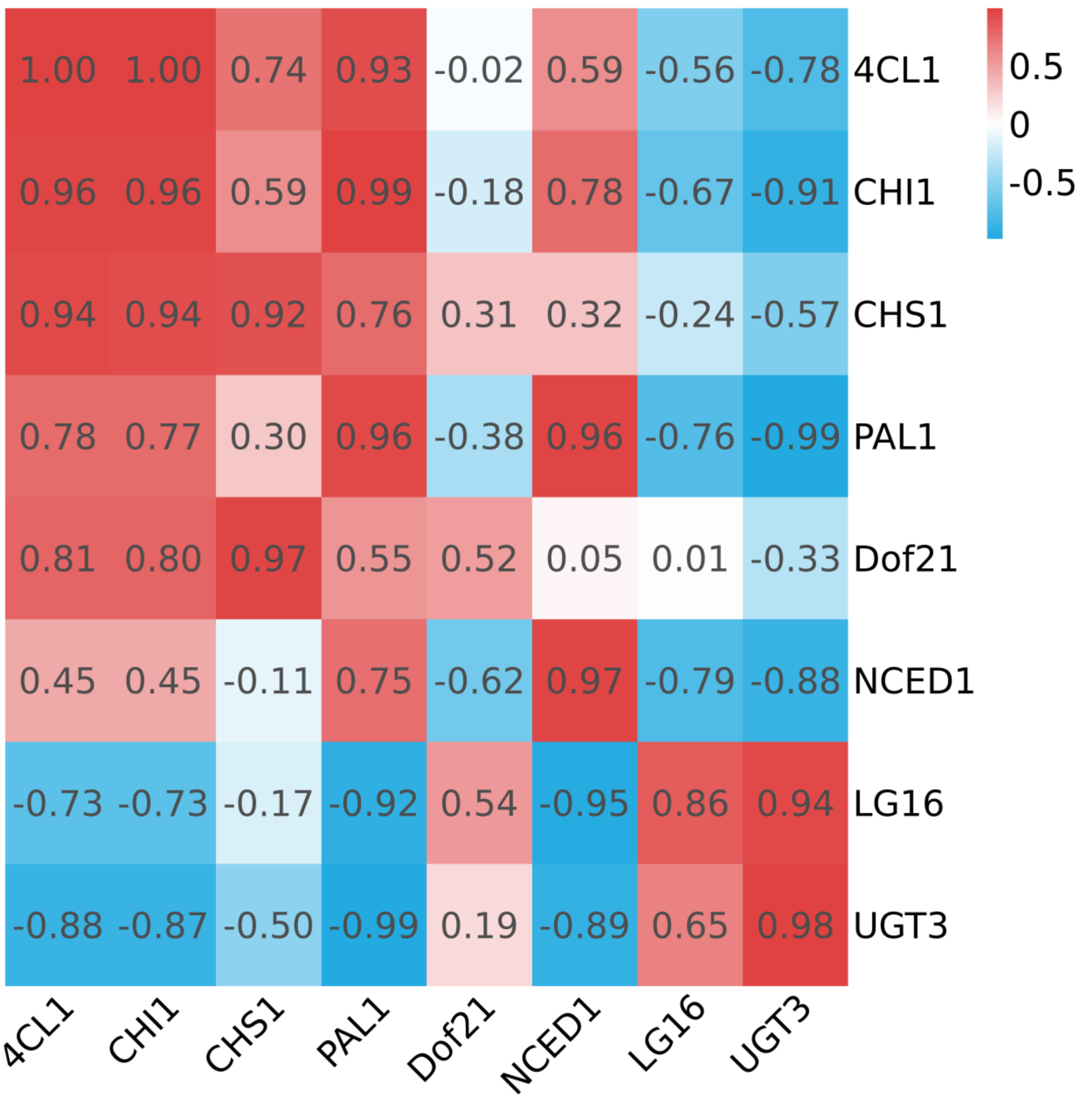

Figure S4. Correlation heatmap between FPKM values and relative expression levels of key DEGs.
